# *De Novo* Transcriptome Analysis of *Oncomelania hupensis* after Molluscicide Treatment by Next-Generation Sequencing: Implications for Biology and Future Snail Interventions

**DOI:** 10.1371/journal.pone.0118673

**Published:** 2015-03-16

**Authors:** Qin Ping Zhao, Tao Xiong, Xing Jian Xu, Ming Sen Jiang, Hui Fen Dong

**Affiliations:** 1 Department of Parasitology, School of Basic Medical Science, Wuhan University, Wuhan, Hubei Province, China; 2 Institute of Schistosomiasis Control, Hubei Provincial Center For Diseases Control and Prevention, Wuhan, Hubei Province, China; Ecole normale superieure de Lyon, FRANCE

## Abstract

The freshwater snail *Oncomelania hupensis* is the only intermediate host of *Schistosoma japonicum*, which causes schistosomiasis. This disease is endemic in the Far East, especially in mainland China. Because niclosamide is the only molluscicide recommended by the World Health Organization, 50% wettable powder of niclosamide ethanolamine salt (WPN), the only chemical molluscicide available in China, has been widely used as the main snail control method for over two decades. Recently, a novel molluscicide derived from niclosamide, the salt of quinoid-2',5-dichloro-4'-nitro-salicylanilide (Liu Dai Shui Yang An, LDS), has been developed and proven to have the same molluscicidal effect as WPN, with lower cost and significantly lower toxicity to fish than WPN. The mechanism by which these molluscicides cause snail death is not known. Here, we report the next-generation transcriptome sequencing of *O*. *hupensis*; 145,008,667 clean reads were generated and assembled into 254,286 unigenes. Using GO and KEGG databases, 14,860 unigenes were assigned GO annotations and 4,686 unigenes were mapped to 250 KEGG pathways. Many sequences involved in key processes associated with biological regulation and innate immunity have been identified. After the snails were exposed to LDS and WPN, 254 unigenes showed significant differential expression. These genes were shown to be involved in cell structure defects and the inhibition of neurohumoral transmission and energy metabolism, which may cause snail death. Gene expression patterns differed after exposure to LDS and WPN, and these differences must be elucidated by the identification and annotation of these unknown unigenes. We believe that this first large-scale transcriptome dataset for *O*. *hupensis* will provide an opportunity for the in-depth analysis of this biomedically important freshwater snail at the molecular level and accelerate studies of the *O*. *hupensis* genome. The data elucidating the molluscicidal mechanism will be of great benefit in future snail control efforts.

## Introduction

The freshwater snail *Oncomelania hupensis* is the only intermediate host of *Schistosoma japonicum*, the agent of the most virulent form of schistosomiasis [1, 2], which is endemic mainly in China and, to a lesser extent, in Indonesia and the Philippines. In China, continuous control efforts have achieved a remarkable reduction in the prevalence of the infection and the burden of disease among humans, making China one of the most successful countries in terms of schistosomiasis control in the world [3–5]; however, the intermediate host *O*. *hupensis* is still present in numerous areas covering a total of 3.69 billion m^2^ [6], mainly in the lake and marshland regions along the Yangtze River. In addition, as the ecology of central and southern China are changing drastically as a result of the construction of the Three Gorges Super Dam, the South-to-North water-transfer project and climate change, new habitats have arisen, and the distribution of *O*. *hupensis*, and thus the distribution and transmission of schistosomiasis, have changed [7–10]. As a consequence, the infection risk to humans and livestock is severe and unpredictable. As schistosomiasis is a neglected tropical disease mainly governed by the geographical distribution of *O*. *hupensis*, snail control, as a major component of the schistosomiasis control program, is still key in attempts to interrupt the transmission of the disease [11, 12] and may be an important component of an integrated strategy for the elimination of schistosomiasis [7, 13, 14].

Although many approaches, including environmental modification, molluscicide treatment, biological control and social intervention, have been used to control the intermediate host snails in China, the best, most cost-effective approach is still not clear [7, 14]. Molluscicide treatment remains the most widely used method due to its wide coverage, simple procedure and fast action. As niclosamide is the only molluscicide recommended by the World Health Organization [15–17] due to its high efficiency, low toxicity and comparatively low environmental contamination, 50% wettable powder of niclosamide ethanolamine salt (WPN) has been extensively used as the only chemical molluscicide in fields in China with endemic schistosomiasis for more than 2 decades. Although WPN has been repeatedly used for long periods, the molluscicidal mechanism is still not well known. Some histochemical studies have shown that WPN immersion can decrease the activity of enzymes in snails, such as phenol oxidase [18], cytochrome C oxidase (CCO), lactate dehydrogenase (LDH), succinate dehydrogenase (SDH), cholinesterase (CHE) and nitric oxide synthase (NOS) [19], leading to hepatopancreas and cerebral ganglion cell damage [20]. These findings suggest that WPN might kill snails by blocking nervous system transmission and metabolic pathways [21]. All of these studies are limited to several enzymatic activities and organ-structure changes in snails after molluscicide treatment. The molecular basis of molluscicidal action is not yet known, hindering identification of valuable target genes and pathways. Park et al. found that niclosamide can cause mitochondrial fragmentation and may contribute to the apoptotic and autophagic cell death of HeLa cells [22], which has yet not been confirmed in snails.

In addition, studies have shown that WPN application has physical complications that include rapid precipitation and difficult dispersion, as well as complications such as toxicity to aquatic animals and expense. To overcome the precipitation and dispersal problems without reducing molluscicidal activity, larger amounts of WPN must be used in the field, leading to increased cost and environmental toxicity [23, 24]. Dai et al. reported a new molluscicidal formulation of niclosamide, a 25% suspension concentrate of niclosamide mixture (SCN) containing niclosamide, alkyl polyglycosides, glycerol sodium carboxymethyl cellulose and sodium benzoate [25]. SCN has improved molluscicidal efficiency, with better dispersion, lower cost and less acute toxicity to fish than WPN; however, its biodegradability [26], effect on chromosomal aberrations and genotoxicity[27] remain to be evaluated. Because niclosamideis currently the only chemical available for snail control, the possibility of the emergence of resistance to this compound in the intermediate host snails has received considerable attention. Although resistance has not been demonstrated [12, 28], the response of *O*. *hupensis* snails to niclosamide has exhibited regional variation in China [12, 28] which make the resistance development possible. In our previous study, a new molluscicide derived from niclosamide, the salt quinoid-2', 5-dichloro-4'-nitro-salicylanilide (Liu Dai Shui Yang An, LDS), was developed and shown to have the same molluscicidal effects as WPN, with lower cost, significantly lower toxicity to fish and a simpler production process [29]. LDS was then recommended as a new choice of molluscicide for use in a variety of habitats. Further studies are required on the molluscicidal mechanism, toxicity and differential effects on protein expression of LDS and WPN to maximize the efficacy of molluscicidal treatment and reduce environmental pollution and the possibility of the emergence of niclosamide resistance.

The objective of this study was to present the first known large-scale transcriptomic dataset generated from *O*. *hupensis* using Illumina technology, with emphasis on the gene-expression profiles of *O*. *hupensis* after treatment with the molluscicides LDS and WPN. The assembled and annotated sequences and gene-expression profiles will allow us to identify most of the genes expressed in *O*. *hupensis* without prior genome information and discover valuable resources for the analysis of the genes and metabolic pathways involved in the molluscicidal mechanism.

## Materials and Methods

### Snail Collection and Molluscicide Assay

*O*. *hupensis* specimens were collected in September 2012 in a location (30°08′ N, 112°22′ E) in Jiangling County, Hubei Province, China. This zone is a marshland beside the Yangtze River and is not a national park or other protected area or private land. Snail control in this zone is supervised by the Institute of Schistosomiasis Control of Jinzhou County, Hubei Province, China. Our collection was permitted by and carried out with the assistance of the institute. No other specific permission was required for us to collect and use these snails. *O*. *hupensis* is not on the list of endangered or protected species. A total of 3,000 snails were brought back to the laboratory. To ensure compatibility, after they were rinsed in dechlorinated tap water and kept for 24 h in the lab, snails with higher vitality ranging from 6 to 8 mm in shell length were selected and then maintained on wet filter paper in a Petri dish. The Petri dish was covered with plastic mesh and placed in an incubator at 25°C that was supplied with fresh humidified air, and the filter paper was changed every three days.

The molluscicidal effects of LDS and WPN (LDS and WPN were obtained from Hubei Provincial Center for Diseases Control and Prevention) were assessed by immersion of the snails in these substances at room temperature (25 ± 1°C). In previous molluscicide experiments, LDS and WPN with active concentrations from 0.05 mg/L to 1 mg/L have been used to estimate molluscicidal activity. More than 50% of the snails died after 12 h treatment, and 100% of the snails died after 24 h treatment at a concentration of up to 0.2 mg/L. To ensure that enough samples could be collected for next-generation sequencing under effective molluscicide treatment, active concentrations of 0.1 mg/L LDS and WPN were used. Twenty active, mature *O*. *hupensis* were exposed to 80 mL in individual 100 mL flasks. In the control group, the same number of snails was kept in dechlorinated tap water under the same conditions. After immersion in LDS, WPN and H_2_O for 6 h, 12 h, 24 h and 48 h, separately, the snails were washed with dechlorinated water, mortality was assessed, and the snails were then crushed for the determination of the infections status [30]. All treatments were performed in triplicate and survival analysis (Kaplan-Meier/Log Rank (Mantel-Cox)) in IBM SPSS Statistics 20 was used to estimate the survival time of snails in immersion test.

The living snails from the molluscicide assay were further washed in diethylpyrocarbonate (DEPC)-treated water. The soft bodies of the snails were dissected out individually on glass slides and examined microscopically to verify infection. The snails that were confirmed to have no helminthic infection were used in this study. The whole soft tissue of each individual was carefully washed 3 times in ice-cold DEPC-treated water containing 0.3% NaCl and then pooled into each group before storage in liquid nitrogen until further use.

### RNA Extraction, mRNA-seq Library Construction and Illumina Sequencing

Total RNA was extracted from 20 living snails for each group separately using TRIzol Reagent (Ambion, Life Technologies) according to the manufacturer’s instructions. Any trace genomic DNA was removed by treating RNA samples with DNase I (Fermentas) prior to cDNA synthesis. The integrity and size distribution of RNA were then verified using an Agilent 2100 Bioanalyzer. RNA samples with RNA integrity numbers (RIN) ≥7.5 were used for cDNA library preparation. The concentration of RNA in each sample was determined using a NanoDrop 2000 spectrophotometer.

The cDNA library was prepared for each group according to the Illumina protocol. In brief, poly (A) mRNA was purified from the total RNA using oligo (dT) magnetic beads and chopped into short fragments using divalent cations under elevated temperature. The cleaved RNA fragments were reverse transcribed into first-strand cDNA using random primers and then synthesized into double-stranded cDNA. From the cDNA, a paired-end library was synthesized for each group using the mRNA-Seq Sample Preparation Kit (Illumina) according to the manufacturer's instructions. Short fragments were purified with the QIA quick PCR Purification Kit (Qiagen), which was also used for continued end repair and ‘A’ base addition. These DNA fragments were then ligated into adapters and purified through gel separation. Finally, the adaptor-ligated libraries were amplified by PCR for sequencing. Illumina sequencing was performed at Beijing Berry Genomics Co., Ltd., using the HiSeq 2000 platform. The transcriptome datasets are available from the NCBI Sequence Read Archive (SRA) under accession number SRP041729.

### Sequence Analysis and Functional Annotation

Raw data generated by Illumina sequencing were transformed by base calling and preprocessed to remove adaptor fragments and low-quality reads to yield clean reads for analysis. These short clean reads were assembled into contigs, transcripts and unigenes using the programs Velvet and Oases. RPKM was calculated by RNA-Seq by Expectation-Maximization (RSEM) program and used to normalize the abundances of the unigenes. The *P* and *Q* value (< 0.05) of expression fold change was calculated in Empirical analysis of digital gene expression data in R (edgeR) and used to identify the unigenes that were differentially expressed between molluscicide-treated groups.

For further analysis, the assembled sequences were searched against the NCBI non-redundant nucleotide database (Nt), non-redundant protein database (Nr) and Swiss-Prot database with an E-value of < 10^−5^. Gene names were assigned to each protein sequence based on the best BLAST hit. To obtain the Gene Ontology (GO) annotation of unigenes, the Blast2GO program was used. WEGO software was used to perform GO functional classifications for all unigenes and to explore the macro-distribution of gene functions. The metabolic pathways of the unigenes were predicted by Kyoto Encyclopedia of Genes and Genomes (KEGG) mapping.

### Validation of Differential Gene Expression

Eleven unigenes that were differentially expressed and two unigenes that were not differentially expressed between the H_2_O-treated group and the LDS-treated group and had significant BLAST hits with the homologs were chosen for validation using real-time PCR with gene-specific primers. Before real-time PCR, semi-quantitative PCR was carried out to confirm the presence or absence of fragments in the original extracted fractions. Total RNA samples were the same as the samples prepared for cDNA library construction. The first-strand cDNAs were random-hexamer primed and synthesized using the RevertAid First Strand cDNA Synthesis Kit (Thermo). Oligonucleotide primers were synthesized based on the sequences determined for differentially expressed candidate genes, and 0.4 μM of each primer was used in the following PCR protocol: 94°C for 3 min followed by 39 cycles of 94°C for 15 s, optimal annealing Tm for 15 s, and 72°C for 20 s. The primers used to amplify the *O*. *hupensis* 18S (F: 5'- CGTCCTTTTGGTGACTCTGG-3'; R: 5'- TGGATGTGGTAGCCGTTTCTC-3') were designed based on the *O*. *hupensis* 18S ribosomal RNA gene partial sequence (AF367667). *O*. *hupensis* 18S was also used in parallel amplification to monitor the transcription of this internal control for normalization. Real-time PCR for each transcript of interest (for primers, see S1 Table) was performed in triplicate in a 20 μL reaction for each reaction, and each reaction was repeated three times, using SYBR Green Real-time PCR master mix (TOYOBO) and a Bio-Rad CFX96 thermal cycler following the manufacturers' protocols. Each of the PCR products was verified by gel electrophoresis as having a clear single band. The mRNA levels were normalized to *O*. *hupensis* 18S, the fold changes were calculated by comparing the mRNA of LDS- and WPN-treated snails to that of H_2_O-treated snails using the comparative delta-delta-Ct method [31], and the results were statistically analyzed using One-way ANOVA and then Post Hoc Multiple Comparisons (Dunnett) in SPSS 13.0.

## Results

### Molluscicide Assay

After the *O*. *hupensis* were immersed in LDS or WPN at an active concentration of 0.1 mg/L or H_2_O for 48 h, snail mortality increased with exposure time both in the LDS- and WPN-treated groups (Table 1, S1 Fig.). The survival time of snails were significant difference (*P* = 0.0000) among three groups by Kaplan-Meier/Log Rank (Mantel-Cox) of survival analysis. By pairwise comparisons (Log Rank (Mantel-Cox)), statistically significant differences were showed in the survival time of LDS-H_2_O paired group (*P* = 0.0000) and WPN-H_2_O paired group (*P* = 0.0000), but no significant difference (*P* = 0.230) in LDS-WPN paired group (Table 1). To sample enough snails in the subsequent experiment, snails that were still living after molluscicide treatment for 12 h were selected.

**Table 1:** Mortality rates (%) of *Oncomelania hupensis* snails after 0.1 mg/L LDS and WPN immersion tests.

Reagent	6 h	12 h	24 h	48 h	Mean survival time (h)*
LDS	8.93 ± 1.56	23.6 ± 1.40	76.05 ± 5.55	88.55 ± 1.85	36.20±1.39**
WPN	5.18 ± 1.38	18.63 ± 2.58	68.4 ± 3.15	78.85 ± 5.15	37.92±1.36**
H_2_O	1.29 ± 0.00	2.17 ± 0.00	2.58 ± 0.00	3.03 ± 0.01	139.68±0.68

* *P* < 0.001, by survival analysis (Kaplan-Meier/Log Rank (Mantel-Cox)) among the three groups.

** By pairwise comparisons (Log Rank (Mantel-Cox)). There are statistically significant differences (*P* < 0.001) in the survival time between LDS- and H_2_O-treated snails, between WPN- and H_2_O-treated snails, but no significant difference (*P* >0.05) between LDS- and WPN-treated snails.

### Transcriptome Sequencing and *De Novo* Assembly

Three pools of cDNA extracted from H_2_O-, LDS- and WPN-treated snails were created and sequenced. After the removal of adaptor sequences, ambiguous reads and low-quality reads, a total of 51,656,919 reads comprising 10,331,383,800 bases from the control (H_2_O-treated) group, 42,375,909 reads comprising 8,475,181,800 bases from LDS treated-snails and 50,975,839 reads comprising 10,195,167,800 bases from WPN-treated snails were obtained. The quality reads from these three libraries were combined and used to generate the transcriptome of *O*. *hupensis*. In total, 254,286 unigenes were obtained with a minimum length of 201 bp, a maximum length of 22,915 bp, and an average length of 670 bp, with 43.27% GC content and an N50 of 1,003 bp. The unigenes ranging from 200 bp to 1,000 bp in length accounted for 83.15% (211,455 unigenes) of the total; 11.12% (28,268 unigenes) were 1,000–2,000 bp long, and 3.55% (9,024 unigenes) were longer than 2,500 bp. Based on FPKM, 234698 unigenes, 222756 unigenes and 228859 unigenes can be mapped in transcriptome of H_2_O-, LDS- and WPN-treated snails, respectively.

### Functional Annotation

**Fig 1:**
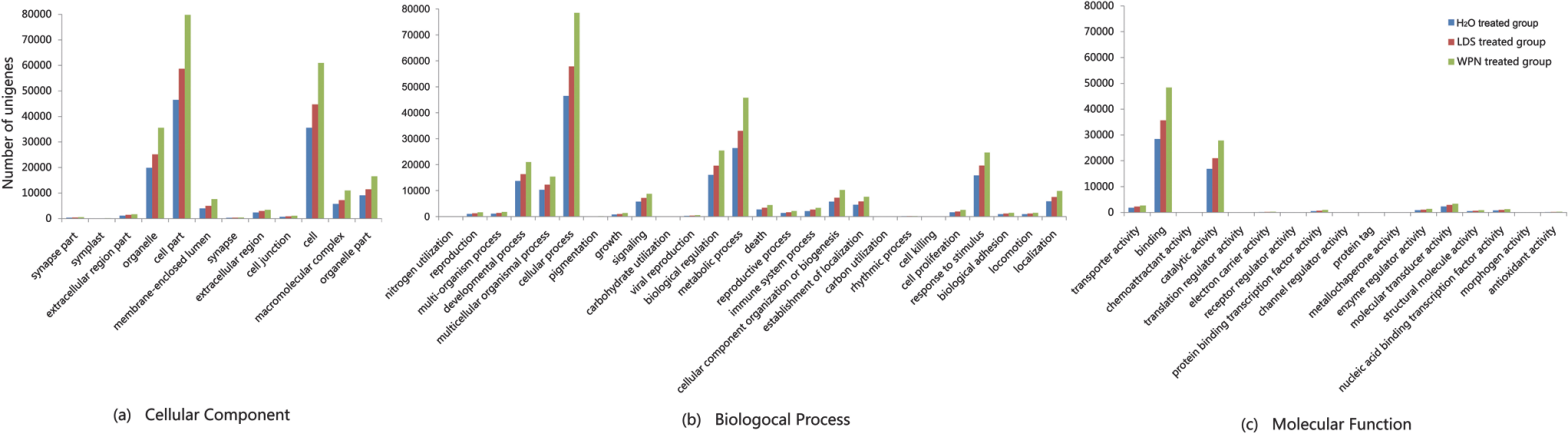
Graphical representation of GO classification of the *O*. *hupensis* putative proteins predicted from the sequencing of unigenes. The unigenes from the H_2_O-, LDS- and WPN-treated groups were classified into three main categories: Cellular Components, Biological Process and Molecular Function. The x-axis indicates the subcategories, and the y-axis indicates the number of unigenes.

Of the all obtained unigenes, 47,906 (18.84%) had significant BLAST hits to homologs in one or more databases (Nt, Nr or Swiss-Prot), with size ranging from 201 bp to 22,915bp, and an average length of 1445 bp. For most of unigenes which a blast hit is unavailable, the length of unigenes ranged from 203 bp to 10,124 bp, with an average length of 490 bp, but all of them contained protein-coding regions with the analysis of GETORF in EMBOSS 3.0. GO analysis was carried out on the putative proteins blasted against the Swiss-Prot database. Of the 35,198 unigenes that could be annotated as putative proteins in Swiss-Prot, 14,860 were assigned one or more GO terms. For the control (H_2_O-treated) group, of 21,620 unigenes matching putative proteins, 8,927 unigenes were assigned one or more GO terms. Of 26,996 and 34,331 unigenes matching putative proteins in the LDS- and WPN-treated groups, 11,173 and 14,545 unigenes were assigned one or more GO terms, respectively (Table 2). All GO assignments fell into broad categories for all three major GO functional domains (Cellular Components, Molecular Function and Biological Process), as presented in Fig. 1. The subcategories in each group are organized in similar patterns. Most of the unigenes in the Cellular Components category were classed as "cell part", "cell" and "organelle"; "binding" and "catalytic activity" dominated in the Molecular Function category. The Biological Process category was composed primarily of "cellular process," followed by "metabolic process," "biological regulation" and "response to stimulus".

**Table 2:** Summary statics of the transcriptome sequencing and bioinformatic analysis for *O*. *hupensis* from the control group (H_2_O group) and molluscicide-treated (LDS and WPN) group.

Distribution and annotation of unigenes	Result
1. Number of total assembled unigenes	254,286
2. Number of unigenes with significant BLAST hits	47,906 (18.84%)
2.1. Hits to Nt	47,526 (18.69%)
2.2 Hits to Nr	6,773 (2.66%)
2.3 Hits to Swiss-Prot (putative proteins)	35,198 (13.84%)
2.3.1 H_2_O-treated group	21,620 (8.50%)
2.3.2 LDS-treated group	26,996 (10.62%)
2.3.3 WPN-treated group	34,331 (13.50%)
3. Unigenes assigned GO terms in total	14,860 (5.84%)
3.1 Matched in different groups:	
3.1.1 H_2_O-treated group	8,927 (3.51%)
3.1.2 LDS-treated group	11,173 (4.39%)
3.1.3 WPN-treated group	14,545 (5.72%)
3.2 Classified into different categories:	
3.2.1 Cellular Components	14,133 (5.56%)
3.2.2 Biological Process	13,383 (5.26%)
3.2.3 Molecular Function	13,953 (5.49%)
4. Number of biological pathways predicted (KEGG)	4,686 (1.84%)

The given number in front of each line represented the classification level.

**Fig 2:**
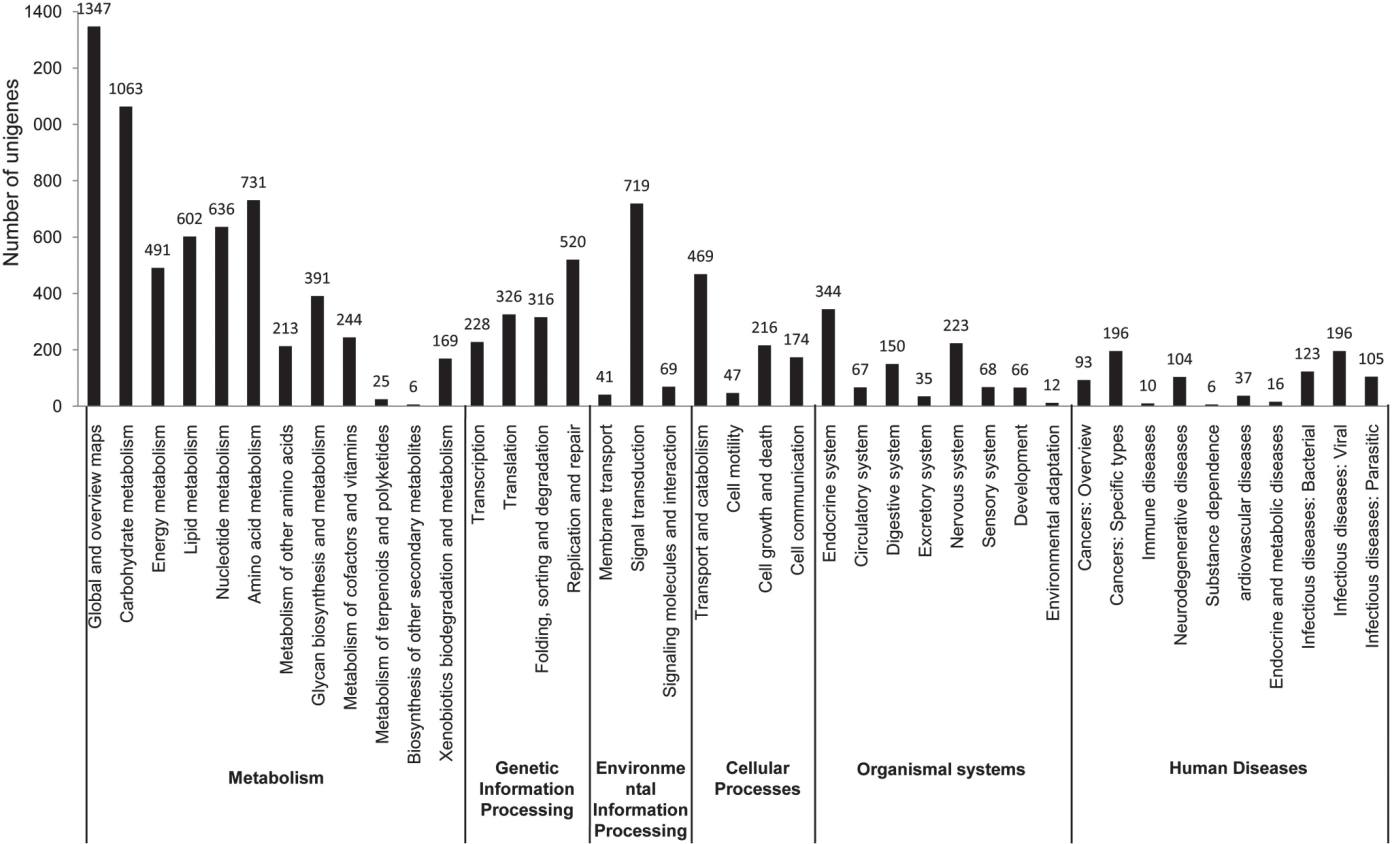
Distribution of the KEGG pathways. Putative proteins were mapped to reference canonical pathways in the KEGG database. Out of the 35,198 putative proteins, 4,686 unigenes were assigned to KEGG pathways among six categories and 41 subcategories. The number of unigenes matched is shown for different pathway subcategories.

KEGG analysis demonstrated the biological pathways in which unigenes are involved. A total of 4,686 unigenes of the 35,198 putative proteins were mapped to 250 KEGG pathways (S2 Table) in six categories and 41 subcategories (Fig. 2). Of the 250 KEGG pathways, there were 89 pathways involved in metabolism, 22 pathways involved in genetic information processing, 56 pathways involved in organismal systems, 15 pathways involved in cellular processes, 18 pathways involved in environmental information processing and 50 pathways involved in human diseases. Among these categories, "metabolic pathways" (1,311 unigenes; 27.98% of KEGG-annotated unigenes), "purine metabolism" (357 unigenes; 7.62% of KEGG-annotated unigenes), "pyrimidine metabolism" (279 unigenes; 5.95% of KEGG-annotated unigenes) and "oxidative phosphorylation" (200 unigenes; 4.27% of KEGG-annotated unigenes) were dominant (S2 Table). The gene catalog provided a comprehensive understanding of the gene-transcription profiles of *O*. *hupensis* and a valuable foundation for screening differentially expressed genes after treatment with molluscicides.

### Differentially Expressed Unigenes Involved in the Response to Molluscicide Exposure

**Fig 3:**
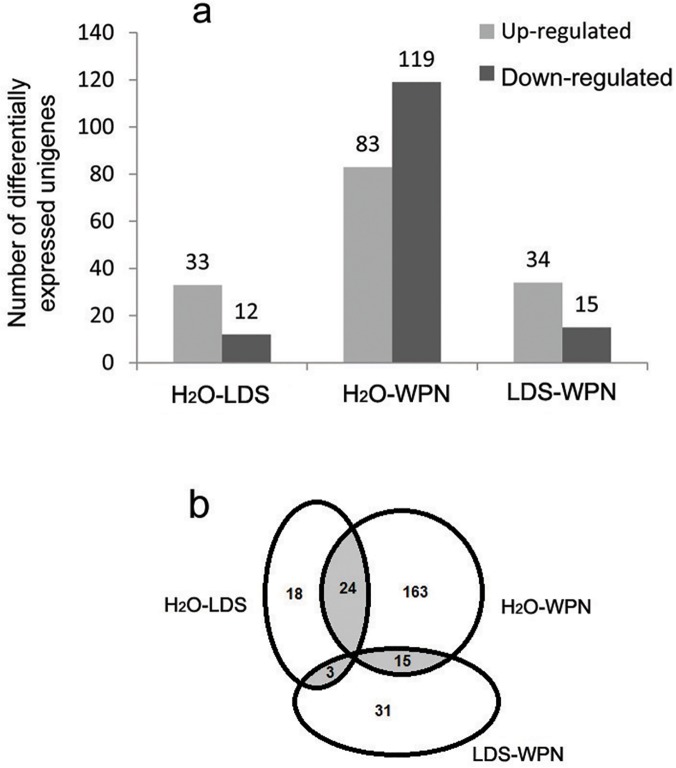
Overview of the distribution of differentially expressed unigenes. a: Comparison of up- and down-regulated unigenes in pairwise groups. For example, for H_2_O-LDS, the former was considered as the control and the latter was considered as the treatment. b: Overlap between the number of differentially expressed unigenes in pairwise groups. The overlapped unigenes are shown in gray. H_2_O: *O*. *hupensis* exposed to H_2_O for 12 h; LDS: *O*. *hupensis* exposed to LDS for 12 h; WPN: *O*. *hupensis* exposed to WPN for 12 h.

To investigate changes in gene expression and identify the critical genes in the *O*. *hupensis* response to molluscicide treatment, the expression levels of unigenes in each library were calculated using the RPKM algorithm. The RPKM value can be used directly to compare the differences in gene expression among samples. The frequency of the RPKM distribution in three samples has a similar pattern (S2 Fig.); most of unigenes belong to RPKM interval (0, 10), and the RPKM interval (90, 100) is the minimum frequency for each sample. A Q value (justified *P* value) ⩽ 0.05 was used as a threshold to evaluate the significance of differences in unigene expression. According to quantification analysis (S3–S5 Tables), a total of 254 unigenes showed significantly differential expression between any pair, whereas no unigene showed a significant difference among all three groups. The amounts of differentially expressed unigenes with each molluscicide treatment are presented in Fig. 3A. Among these unigenes, compared to the H_2_O-treated group, 33 and 12 unigenes were up-regulated and down-regulated at 12 h after LDS exposure, respectively, and 83 and 119 unigenes were up-regulated and down-regulated at 12 h after WPN exposure, respectively. Forty-two unigenes overlapped among differentially expressed unigenes of paired groups (Fig. 3B). Among the 24 overlapping unigenes between H_2_O-LDS and H_2_O-WPN, 17 unigenes were up-regulated after both LDS and WPN exposure compared to the H_2_O-treated group, and 7 unigenes were down-regulated in both cases. For 15 overlapping unigenes between H_2_O-WPN and LDS-WPN, 9 were up-regulated and 6 were down-regulated after WPN exposure in both paired groups, while no different expression for these unigenes between H_2_O- and LDS-treated group. For 3 overlapping unigenes between H_2_O-LDS and LDS-WPN, all of them were up-regulated after LDS exposure compare to H_2_O and WPN exposure, with no different expression between H_2_O- and WPN-treated group.

**Fig 4:**
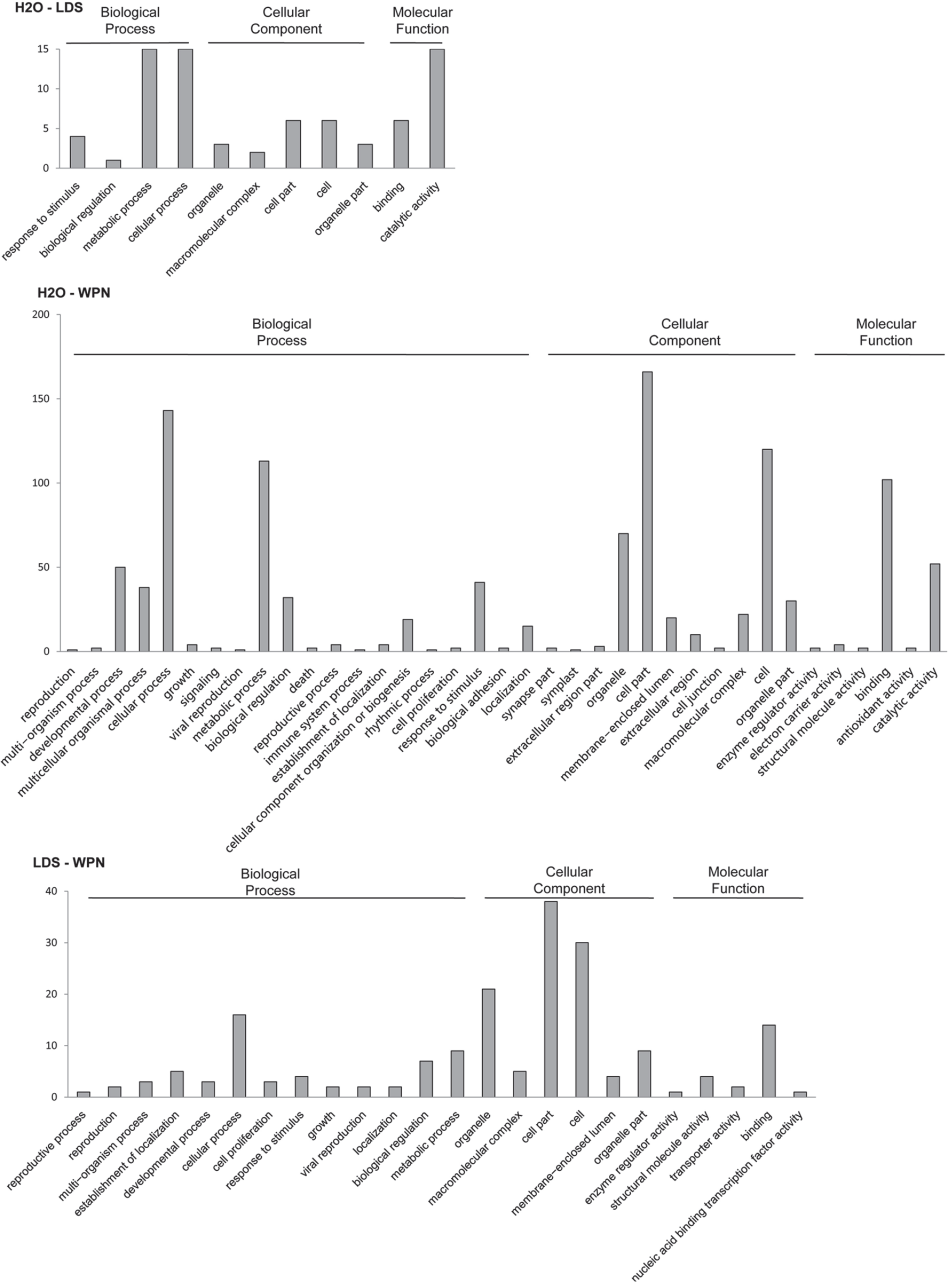
Graphical representation of the GO classification of unigenes differentially expressed between pairwise groups. All GO terms of unigenes in each pairwise group were classified into either the Cellular components, Biological Process or Molecular Function category. The x-axis indicates the subcategories, and the y-axis indicates the number of unigenes involved (as one unigene can be annotated as several terms in each GO category, the number of unigenes here represents the number of times that unigene was involved).

To understand the functions of differentially expressed unigenes, GO functional and KEGG pathway analyses were performed for each paired group, although the number of unigenes that could be annotated was limited. For 45 unigenes differentially expressed between the H_2_O-treated and LDS-treated groups, 12 unigenes had significant BLAST hits with homologs in one or more databases (Nt, Nr or Swiss-Prot). Among these unigenes, 10 were annotated as putative proteins in Swiss-Prot, two were assigned one or more GO terms, and three were mapped to KEGG pathways. Of the 202 differentially expressed unigenes in the H_2_O-WPN paired group, 105 unigenes had significant BLAST hits with homologs in one or more databases and 88 were annotated as putative proteins in Swiss-Prot. Among these 88 unigenes, 27 were assigned GO terms and 13 unigenes were mapped to KEGG pathways. Thirty-eight out of 49 unigenes differentially expressed between the LDS- and WPN-treated groups had significant BLAST hits to homologs in known databases. Six of these 38 unigenes were assigned GO terms, and seven were mapped to KEGG pathways (S6 Table). When comparing all GO terms of differentially expressed genes in the pairwise groups, "cellular process" and "metabolic process" were the most frequently enriched terms involved in Biological Process for any pairwise group, namely, H_2_O-LDS, H_2_O-WPN and LDS-WPN. "Cell part" and "cell" were the most frequently enriched terms involved in Cellular Components for any pairwise group. For GO terms classified as Molecular Function, the most frequently enriched terms were associated with "binding" and "catalytic activity", although no unigene involved in "catalytic activity" was enriched in LDS-WPN (Fig. 4).

### Validation of Illumina Expression Patterns by qRT-PCR

**Fig 5:**
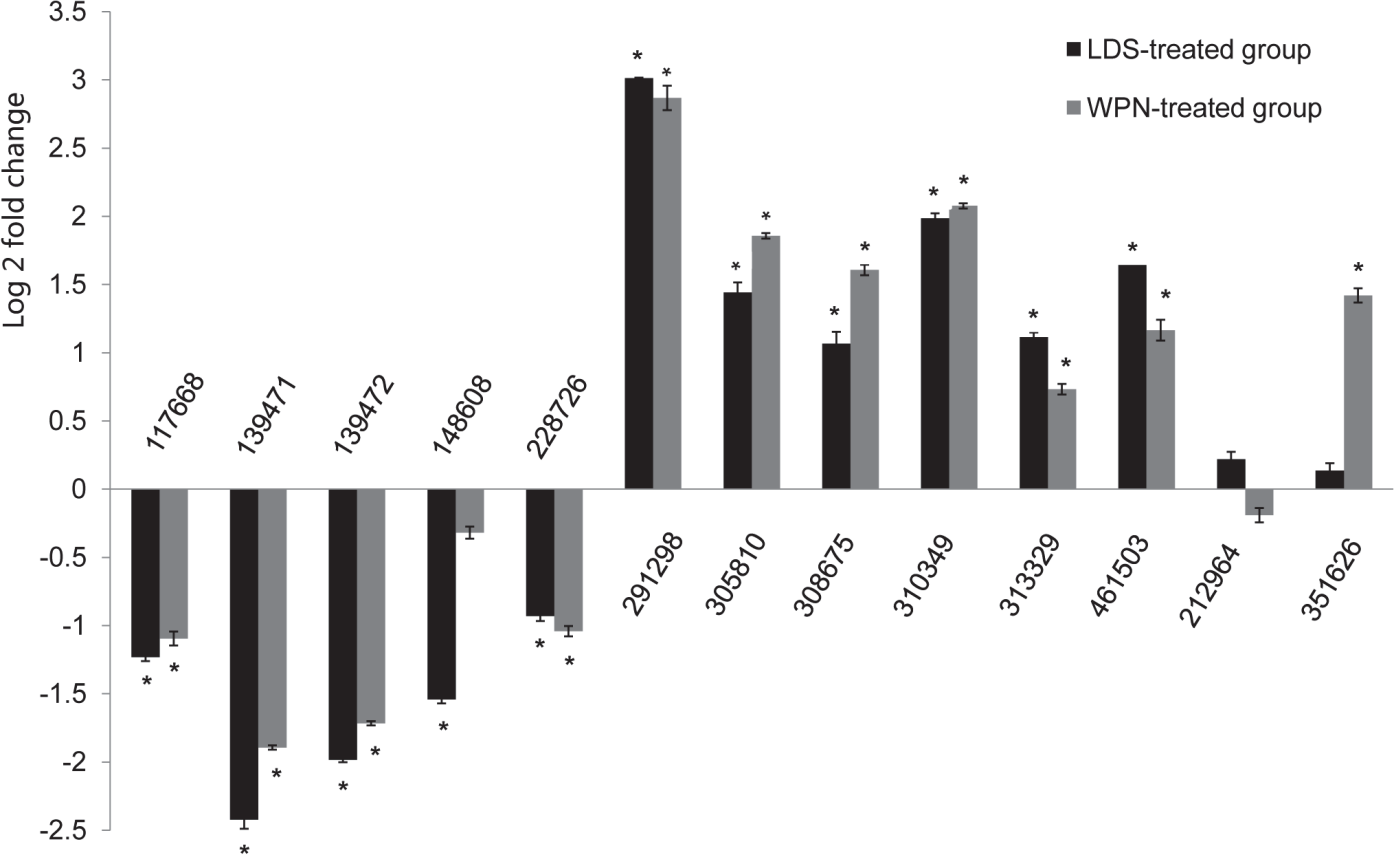
Expression profiles of 11 selected differentially expressed unigenes and 2 unigenes with no different expression among H_2_O-treated, LDS-treated and WPN-treated groups evaluated by real-time PCR. mRNA-expression values in H_2_O-treated snails were calculated as the control for each unigene and showed with log2 (fold change) on the Y axis. * at the top of each error bar represents *p* values of < 0.05, as calculated with One-way ANOVA and then Post Hoc Multiple Comparisons in SPSS.

To confirm the reliability of the sequencing analysis and to verify the relationship of the unigenes after molluscicide treatment, 13 unigenes in the H_2_O-LDS pairwise group were selected and detected by qRT-PCR, including 11 differentially expressed unigenes and 2 unigenes that showed no significant difference in sequencing. Before qRT-PCR, all candidates were detected in *Oncomelania* cDNA and showed a band of the expected size through agarose-gel electrophoresis by semi-quantitative PCR. The profile of their expression (Fig. 5) was consistent with the results of the sequencing. The expression of two unigenes with no different expression in sequencing also showed no significant difference after LDS and WPN exposure in real-time PCR, with the exception of unigene 351626, which were up-regulated after WPN exposure, and the expression level was significantly different, consistent with the sequencing result. Five of those 11 differentially expressed unigenes were down-regulated, and six of those 11 unigenes were up-regulated after LDS or WPN exposure, and the expression levels were both significantly different compared to the H_2_O-treated group, with the exception of unigene 148608, which showed no significant difference in the H_2_O-WPN pairwise group, consistent with the sequencing result. Although unigene 313329 showed no significant difference (*Q* = 0.078) in expression level between H_2_O and WPN by sequencing, the difference was confirmed to be significant by real-time PCR (*P* = 0.007) and transcriptome sequencing (*P* = 4.53×10^–5^).

## Discussion

According to the immersion assay, the molluscicidal activity of LDS against *O*. *hupensis* at an active concentration of 0.1 mg/L showed no significant difference compared to that of WPN, confirming previous results of lab and field assays by Yuan et al. [29]. The major difficulties in using molluscicides to control snails are the cost and the toxicity to fish. Previous studies imply that LDS has the least acute toxicity to fish among the molluscicides accepted for use by the Chinese government [25, 29]. In addition, based on its application in 23 counties in 8 provinces, LDS has 13.17–36.84% lower costs than WPN (data not shown). LDS is thus strongly recommended as a new choice of molluscicide. It is necessary to understand the molluscicidal mechanism of LDS and determine the difference between LDS and WPN, which will have implications for identifying potential key molecules related with metabolism, apoptosis and cell death after chemical treatment. This will help us to understand metabolic pathways or apoptotic programs which may be vital to snail development and death, to snail control finally.

With continued computational developments, high-throughput mRNA sequencing technology with *de novo* assembly is especially suitable for gene-expression profiling in those species that do not have closely related reference genomes [32, 33]. Here, by profiling the transcriptome of *O*. *hupensis* on the Illumina Hiseq 2000 platform, 29 Gb of coverage with 145,008,667 clean reads was obtained and assembled into 254,286 unigenes by *de novo* assembly. This work provided the largest genomic dataset for *O*. *hupensis*, the first extensive genetic resources for this biomedically important freshwater snail, for which very limited genetic or genomic information was available in public databases. Previously available data included 1,432 EST sequences from an *O*. *hupensis* hepatopancreas cDNA library [34] and head-foot SSH-library [35], partial and complete mitochondrial sequences [36], several internal transcribed spacer sequences[37], ribosomal RNA genes and microsatellites [38, 39]. Due to the small proportion of mollusk-specific sequences in current databases, only 18.84% (47,906 unigenes) of 254,286 unigenes were successfully matched to homologs in Nt, Nr or Swiss-Prot. To confirm that the transcriptome dataset presented here corresponds to *O*. *hupensis*, 10,000 reads were randomly selected and analyzed, 1,717 showed significant BLAST hits with homologs in the Nt database, and 38.7% (665 reads) of which most closely matched *O*. *hupensis*.

Based on general GO analysis, 42.2% (14,860 unigenes) of the 35,198 putative protein sequences could be annotated, suggesting that they have relatively conserved functions. Although these sequences comprise a small part of the transcriptome sequences, this identification will certainly improve our understanding of the development of snail organs or tissues, biological regulation and metabolites, stimulus and immune response. Considering the key role of this species in schistosomiasis transmission and control, we focused on the immune-system-related and death-related genes. Many new genes have been identified, including 3,377 unigenes involved in immune-system processes, 790 unigenes involved in apoptosis and 374 unigenes involved in cell death. Not only the gene or protein names and descriptions but also their putative conserved domains, GO terms, and potential metabolic pathways were assessed. For example, 48 unigenes were identified as toll-like receptors (TLRs) in this study, with high identities to TLR1-4, TLR6, TLR8, TLR13 and TLR22 from oysters to humans (data not shown); most of these TLRs have a conserved structure, including leucine-rich repeats (LRR), trans-membrane (TM) regions and a Toll-Interleukin 1 receptor (TIR) domain. In addition, 5 unigenes were identified as MyD88, and several unigenes showed high identities to TRAF6, TRAF3, IRAK4, TBK1, NFκB1 and NFκB2. All of these molecules were confirmed to be involved in the TLR signaling pathway [40], which plays a central role in the initiation of innate immunity, including host-cell recognition and response to pathogens [41]. These findings from transcriptome analysis will be beneficial to understanding the role of TLRs in inducing innate immune responses and in shaping the adaptive immunity of freshwater snails. KEGG predictions allowed us to identify 13.3% (4,686 unigenes) of the 35,198 putative proteins mapped to 250 KEGG pathways. Of these 4,686 unigenes, 59 contributed to apoptosis and 41 contributed to TLR signaling. These transcriptome data will provide new insights and facilitate further studies of *O*. *hupensis* genes and their functions.

In sequencing transcripts from groups treated with different molluscicides, 45 unigenes showed significant differential expression after 12 h of exposure to LDS, of which 33 were up-regulated and 12 were down-regulated; 202 unigenes showed significant differential expression after 12 h of exposure to WPN, of which 83 were up-regulated and 119 were down-regulated. Thus, the gene-expression pattern differed even opposite after exposure to LDS and WPN, as confirmed by the 49 unigenes that were differentially expressed between LDS and WPN exposure. Because the available matched putative proteins were limited, information from GO and KEGG analysis for the differentially expressed unigenes was not sufficient to identify molecules or pathways critical to inducing snail death after molluscicide treatment. However, the unigenes involved in "cellular process" and "metabolic process" showed the most significant differences after LDS and WPN exposure, and unigenes involved in "binding" and "catalytic activity" also showed significant differences after LDS and WPN exposure. This result was consistent with ultrastructural and metabolic enzyme changes observed in snails in our study. After LDS and WPN exposure, the muscle fibers loosened and became disordered with widened gaps, neural fibers severed, cell membranes collapsed, the number of glycogen granules of the endoplasmic reticulum decreased, mitochondria deformed, vacuolization occurred, nuclear membranes ruptured or disappeared, heterochromatin polarized in the hepatopancreas and muscle cells, and the number of endocrine granules decreased and that of secondary lysosomes increased in hepatopancreas cells (data not shown). In addition, the activity of some enzymes showed changes after molluscicide treatment; for example, alanine aminotransferase (ALT), lactate dehydrogenase (LDH) and nitric oxide synthase (NOS) activity decreased dramatically, whereas the activity of superoxide dismutase (SOD) increased followed by decreasing to levels below those in the control group. This result may imply that molluscicides cause defects in cell structure, inhibit neurohumoral transmission and energy metabolism, and cause snail death. The cell defects in LDS-treated snails were more severe than those in the WPN-treated group. This difference is perhaps related with the 49 differentially expressed unigenes, or the opposite regulation pattern on gene expression that require further identification and investigation.

In summary, the first published large-scale transcriptome analysis of *O*. *hupensis*, the only intermediate host of *S*. *japonicum*, is presented here. A total of 145,008,667 clean reads were assembled into 254,286 unigenes. The availability of this transcriptome dataset forms the basis for further studies of the growth, development, death and immune response of *O*. *hupensis*, which will be beneficial for snail control and interruption of disease transmission through biological methods. In total, 254 unigenes showed significantly differential expression after molluscicide treatment. GO and KEGG analyses, as well as morphological analyses, indicated that cell structure defects and the inhibition of neurohumoral transmission and energy metabolism caused by LDS and WPN lead to snail death. These findings will be more fully elucidated after these unigenes have been identified and annotated completely.

## Supporting Information

S1 Fig The survival curve of *Oncomelania hupensis* snails in H_2_O-, LDS- and WPN-treated groups. (TIF)

S2 Fig The frequency of RPKM distribution in H2O-, LDS- and WPN-treated samples. (TIF)

S1 Table Real-time PCR oligonucleotide primers and conditions. (DOCX)

S2 Table Summary of the KEGG pathways of mapped unigenes of *Oncomelania hupensis*. (XLSX)

S3 Table Unigenes differentially expressed between the H2O-treated and LDS-treated groups based on the RPKM value. (XLSX)

S4 Table Unigenes differentially expressed between the H2O-treated and WPN-treated groups based on the RPKM value. (XLSX)

S5 Table Unigenes differentially expressed between the LDS-treated and WPN-treated groups based on the RPKM value. (XLSX)

S6 Table GO terms and KEGG pathways for differentially expressed genes with significant BLAST hits in current databases. (XLSX)

## References

[1] Ross AG, Sleigh AC, Li Y, Davis GM, Williams GM, Jiang Z, et al Schistosomiasis in the People's Republic of China: prospects and challenges for the 21st century. Clin Microbiol Rev. 2001; 14: 270–295. 11292639 10.1128/CMR.14.2.270-295.2001 PMC88974

[2] Shrivastava J, Qian BZ, McVean G, Webster JP. An insight into the genetic variation of *Schistosoma japonicum* in mainland China using DNA microsatellite markers. Mol Ecol. 2005; 14: 839–849. 15723675 10.1111/j.1365-294X.2005.02443.x

[3] Zhou XN, Wang LY, Chen MG, Wu XH, Jiang QW, Chen XY, et al The public health significance and control of schistosomiasis in China—then and now. Acta Trop. 2005; 96: 96–105. 10.1016/j.actatropica.2005.07.005 16125655

[4] Bergquist R, Tanner M. Controlling schistosomiasis in Southeast Asia: a tale of two countries. Adv Parasitol. 2010; 72: 109–144. 10.1016/S0065-308X(10)72005-4 20624530

[5] Liu R, Dong HF, Jiang MS. The new national integrated strategy emphasizing infection sources control for schistosomiasis control in China has made remarkable achievements. Parasitol Res. 2013; 112: 1483–1491. 10.1007/s00436-013-3295-5 23354940

[6] Li SZ, Zheng H, Gao J, Zhang LJ, Zhu R, Xu J, et al Endemic status of schistosomiasis in People's Republic of China in 2012. Chin J Schisto Control. 2013; 25: 557–563. 24490385

[7] Yuan Y, Xu XJ, Dong HF, Jiang MS, Zhu HG. Transmission control of schistosomiasis japonica: implementation and evaluation of different snail control interventions. Acta Trop. 2005; 96: 191–197. 16154105 10.1016/j.actatropica.2005.07.014

[8] Seto EY, Wu W, Liu HY, Chen HG, Hubbard A, Holt A. Impact of changing water levels and weather on Oncomelania hupensis hupensis populations, the snail host of Schistosoma japonicum, downstream of the Three Gorges Dam. Ecohealth. 2008; 5: 149–158. 10.1007/s10393-008-0169-x 18787918

[9] McManus DP, Li Y, Gray DJ, Ross AG. Conquering 'snail fever': schistosomiasis and its control in China. Expert Rev Anti Infect Ther. 2009; 7: 473–485. 10.1586/eri.09.17 19400766

[10] Gray DJ, Li YS, Williams GM, Zhao ZY, Harn DA, Li SM, et al A multi-component intergrated approach for the elimination of schistosomiasis in the People's Republic of China: design and baseline results of a 4-year cluster-randomised intervention trial. Int J Parasitol. 2014; 44: 659–668. 10.1016/j.ijpara.2014.05.005 24929133

[11] Wang L, Utzinger J, Zhou XN. Schistosomiasis control: experiences and lessons from China. Lancet. 2008; 372: 1793–1795. 10.1016/S0140-6736(08)61358-6 18930529 PMC7135384

[12] Dai JR, Li YZ, Wang W, Xing YT, Qu GL, Liang YS. Resistance to niclosamide in Oncomelania hupensis, the intermediate host of Schistosoma japonicum: should we be worried? Parasitology. 2014; 8: 1–9. 10.1017/S0031182014000870 25003984

[13] Zhou XN, Bergquist R, Leonardo L, Yang GJ, Yang K, Sudomo M, et al Schistosomiasis japonica: control and research needs. Adv Parasitol. 2010; 72: 145–178. 10.1016/S0065-308X(10)72006-6 20624531

[14] Yang GJ, Sun LP, Hong QB, Zhu HR, Yang K, Gao Q, et al Optimizing molluscicide treatment strategies in different control stages of schistosomiasis in the People’s Republic of China. Parasit Vectors. 2012; 5: 260 10.1186/1756-3305-5-260 23151396 PMC3533975

[15] WHO. The control of schistosomiasis: report of a WHO expert committee. World Health Organ Tech Rep Ser. 1985; 728: 58–61. 3938106

[16] WHO. The control of schistosomiasis: second report of the WHO expert committee. World Health Organ Tech Rep Ser. 1993; 830: 51–53. 8322462

[17] WHO Expert Committee. Prevention and control of schistosomiasis and soil-transmitted helminthiasis. World Health Organ Tech Rep Ser. 2002; 912: 7–17. 12592987

[18] Yang JS, Zhou SL, Tang XN, Cheng WK. Influence of niclosamide on phenol oxidase activity in *Oncomelania* snails. Chin J Schisto Control. 2007; 19: 311–312.

[19] Li HJ, Liang YS, Dai JR, Xu M, Ru WW, Xu YL. Enzyme-histochemical observation on influence of suspension concentrate of niclosamide in *Oncomelania hupensis* snails. Chin J Schisto Control. 2006; 18: 427–430.

[20] Li WG, Huang SX, Xu MX, Wang YM, Hu LL, Gao ZQ, et al Ultrastructural changes of cerebral ganglion of *Oncomelania hupensis* after immersion in niclosamide. Chin J Parasit Dis Control. 1997; 10: 42–45.

[21] Xing YT, Dai JR. Progress of research on molluscicide niclosamide. Chin J Schisto Control. 2010; 22: 504–508.

[22] Park SJ, Shin JH, Kang H, Hwang JJ, Cho DH. Niclosamide induces mitochondria fragmentation and promotes both apoptotic and autophagic cell death. BMB Rep. 2011; 44: 517–522. 21871175 10.5483/bmbrep.2011.44.8.517

[23] Oliveira-Filho EC, Paumgartten FJ. Toxicity of Euphorbia milii latex and niclosamide to snails and non target aquatic species. Ecotoxicol Environ Saf. 2000; 46: 342–350. 10903832 10.1006/eesa.2000.1924

[24] Zhang T, Jiang QW. Study on toxicology of niclosamide. Chin J Schisto Control. 2002; 14: 234–236.

[25] Dai JR, Wang W, Liang YS, Li HJ, Guan XH, Zhu YC. A novel molluscicidal formulation of niclosamide. Parasitol Res. 2008; 103: 405–412. 10.1007/s00436-008-0988-2 18454287

[26] Graebing PW, Chib JS, Hubert TD, Gingerich WH. Metabolism of niclosamide in sediment and water systems. J Agric Food Chem. 2004; 52: 5924–5932. 15366844 10.1021/jf0401524

[27] Ostrosky-Wegman P, García G, Montero R, Pérez Romero B, Alvarez Chacón R, Cortinas de Nava C. Susceptibility to genotoxic effects of niclosamide in human peripheral lymphocytes exposed in vitro and in vivo. Mutat Res. 1986; 173: 81–87. 3941681 10.1016/0165-7992(86)90015-1

[28] Dai J, Li Y, Wang W, Xing Y, Qu G, Liang Y. Sensitivity of Oncomelania hupensis to niclosamide: a nation-wide survey in China. Int J Environ Res Public Health. 2014; 11: 3086–3095. 10.3390/ijerph110303086 24625624 PMC3987021

[29] Yuan Y, Dong HF, Xu XJ, Li GL, Wei FH, Zhao YB. Evaluation of a new molluscicide for counteracting the intermediate snail host of Schistosoma japonicum. Malacologia. 2011; 53: 217–227.

[30] MOH. Handbook of schistosomiasis control (Ministry of Health) 3rd ed. Shanghai: Shanghai Science and Technology Literature Publishing House; 2000, pp. 204–208.

[31] Livak KJ, Schmittgen TD. Analysis of relative gene expression data using real-time quantitative PCR and the 2 ^(−Delta Delta C(T))^ method. Methods. 2001; 25: 402–408. 11846609 10.1006/meth.2001.1262

[32] Vera JC, Wheat CW, Fescemyer HW, Frilander MJ, Crawford DL, Hanski I, et al Rapid transcriptome characterization for a nonmodel organism using 454 pyrosequencing. Mol Ecol. 2008; 17: 1636–1647. 10.1111/j.1365-294X.2008.03666.x 18266620

[33] Dheilly NM, Adema C, Raftos DA, Gourbal B, Grunau C, Du Pasquier L. No more non-model species: the promise of next generation sequencing for comparative immunology. Dev Comp Immunol. 2014; 45: 56–66. 10.1016/j.dci.2014.01.022 24508980 PMC4096995

[34] Zhu DD, Zhao QP, Nie P. Construction and analysis on cDNA library of Oncomelania hupensis hepatopancreas. Acta Hydrobiol Sin. 2011; 35: 672–680.

[35] Wang H, Zhao QP, Nie P, Jiang MS, Song J. Identification of differentially expressed genes in Oncomelania hupensis chronically infected with Schistosoma japonicum. Exp Parasitol. 2012; 130: 374–383. 10.1016/j.exppara.2012.02.004 22343044

[36] Zhao QP, Zhang SH, Deng ZR, Jiang MS, Nie P. Conservation and variation in mitochondrial genomes of gastropods O*ncomelania hupensis* and *Tricula hortensis*, intermediate host snails of Schistosoma in China. Mol Phylogenet Evol. 2010; 57: 215–226. 10.1016/j.ympev.2010.05.026 20595013

[37] Zhao QP, Jiang MS, Littlewood DT, Nie P. Distinct genetic diversity of *Oncomelania hupensis*, intermediate host of *Schistosoma japonicum* in mainland China as revealed by ITS sequences. PLoS Negl Trop Dis. 2010; 4: e611 10.1371/journal.pntd.0000611 20209150 PMC2830461

[38] Zhang SH, Zhao QP, Jiao R, Gao Q, Nie P. Identification of polymorphic microsatellites for the intermediate host *Oncomelania hupensis* of *Schistosoma japonicum* in China. Malacologia. 2010; 53: 147–153.

[39] Zhang L, Li S, Wang Q, Qian Y, Liu Q, Yang P, et al Isolation and characterization of 15 new microsatellite markers in Oncomelania hupensis, the snail intermediate host of Schistosoma japonicum in mainland China. Int J Mol Sci. 2012; 13: 5844–5850. 10.3390/ijms13055844 22754335 PMC3382821

[40] Rauta PR, Samanta M, Dash HR, Nayak B, Das S. Toll-like receptors (TLRs) in aquatic animals: signaling pathways, expressions and immune responses. Immunol Lett. 2014; 158: 14–24. 10.1016/j.imlet.2013.11.013 24291116

[41] Kawai T, Akira S. Toll-like receptors and their crosstalk with other innate receptors infection and immunity. Immunity. 2011; 34: 637–650. 10.1016/j.immuni.2011.05.006 21616434

