# Supplementary figures and images for: De Novo Transcriptome Analysis of Oncomelania hupensis after Molluscicide Treatment by Next-Generation Sequencing: Implications for Biology and Future Snail Interventions
Source: PLoS One. 2015 Mar 16;10(3):e0118673. doi: 10.1371/journal.pone.0118673 (PMC4361594; doi:10.1371/journal.pone.0118673)

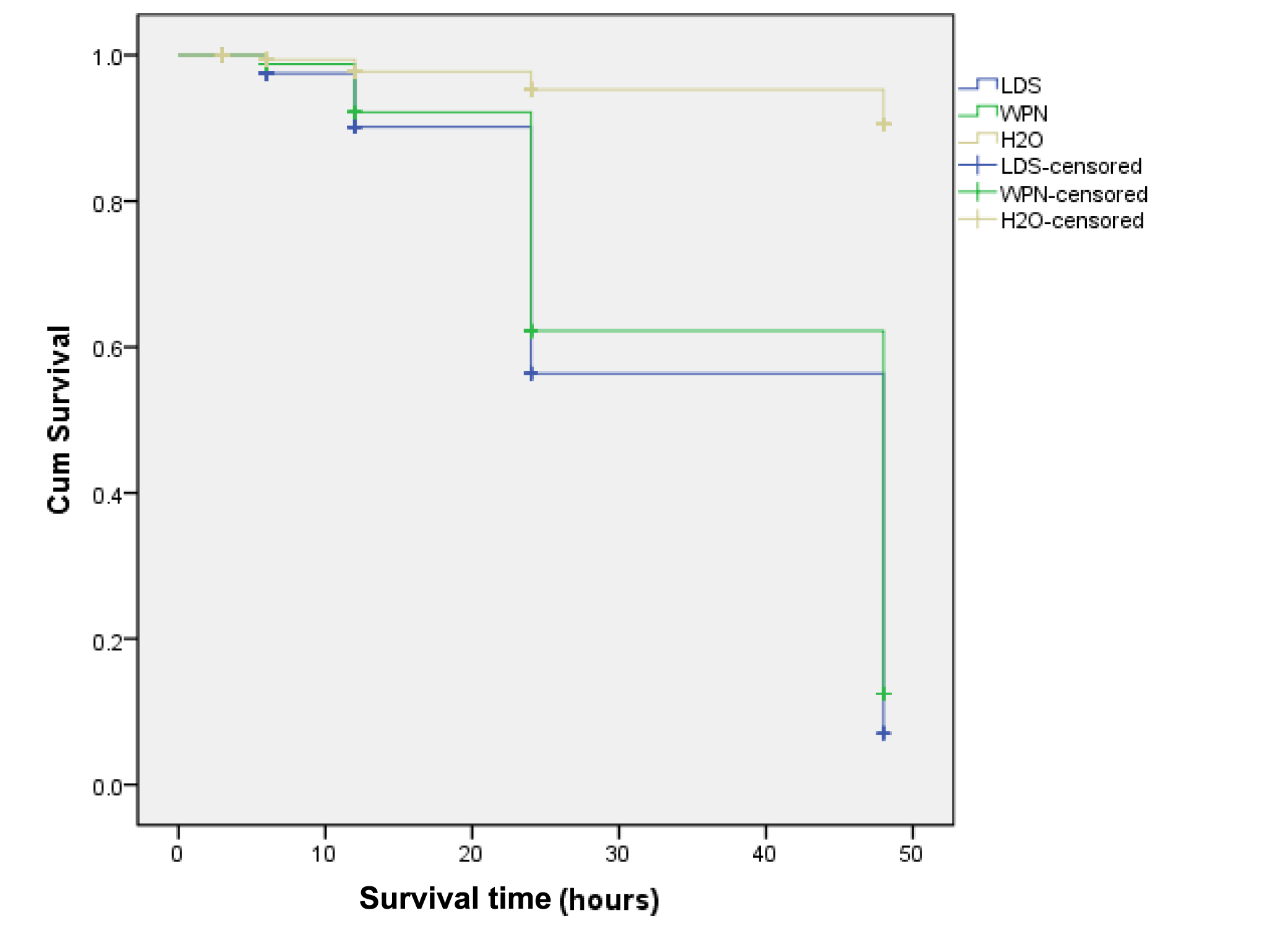

Supplement: S1 Fig — (TIF) [file pone.0118673.s001.tif]

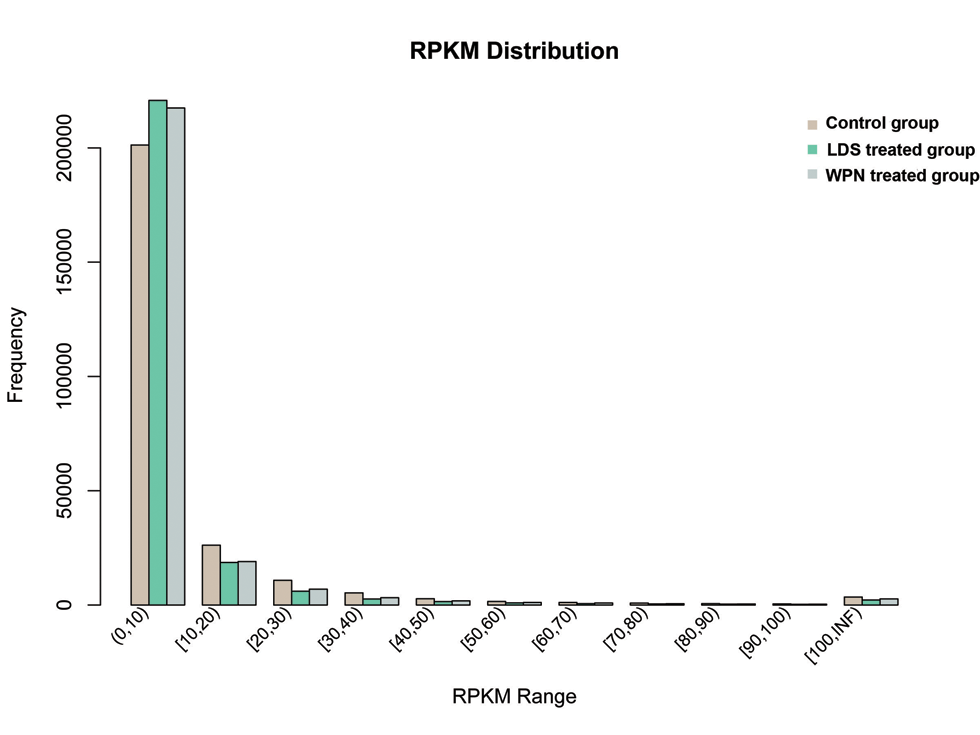

Supplement: S2 Fig — (TIF) [file pone.0118673.s002.tif]
