# Supplementary material for: De Novo Transcriptome Analysis of Oncomelania hupensis after Molluscicide Treatment by Next-Generation Sequencing: Implications for Biology and Future Snail Interventions
Source: PLoS One. 2015 Mar 16;10(3):e0118673. doi: 10.1371/journal.pone.0118673 (PMC4361594; doi:10.1371/journal.pone.0118673)
Supplement: S1 Table — (DOCX) [file pone.0118673.s003.docx]

**Table S1. Real-time PCR oligonucleotide primers and conditions.**

| Unigene no. | Forward / reverse primer (5' - 3') | PCR annealing conditions |
| --- | --- | --- |
| 117668 | 5'- TGTTCTAGCCGCCACCAGT -3'  5'- GAAGCCTAACTCCTACTTTGTC-3' | 54°C / 15 s |
| 139471 | 5' - CTCTGGCTACGATGAACTGAAG - 3'  5' - CGAACGGCAAACTGAGGAAA - 3' | 54°C / 15 s |
| 139472 | 5' - GTCAGGTTGGTAAGGCGAC - 3'  5' - CCAGCGGAATCAAAGGAATAC- 3' | 54°C / 15 s |
| 148608 | 5' - CAAAACCTTCTAATCACCACCTAC- 3'  5' - TCTTCACCGTCAAGAGCAGTA - 3' | 54°C / 15 s |
| 228726 | 5' - CGGAGACTTTTGCCTGCTGTG – 3'  5' - CTTGAGATGGGAACGGTTTGAG - 3' | 57°C / 15 s |
| 291298 (291303) | 5' - CCACGCCGAAATCTCGCATAG - 3'  5' - CAGCAAAGTGGCCGAACTCAC - 3' | 60°C / 15 s |
| 305810 | 5' - GGAGAAAGCGAAGTCAGCAGT - 3'  5' - CACCGAAAGACGACCGCAAAG - 3' | 60°C / 15 s |
| 308675 | 5' - CTCAGCCGCAGCAGTAGACTC - 3'  5' - AGCCGCTCAGTGCCCCATTATC- 3' | 57°C / 15 s |
| 310349 | 5' - CTATCGGAGTTGCTGTGGTGG - 3'  5' - GTTGGTCTGGTGCGAGCCTTT - 3' | 60°C / 15 s |
| 313329 | 5' - GCACAGGGGCAGAAGACGAG - 3'  5' - CTTCACCAGGCATTCCACAGC- 3' | 60°C / 15 s |
| 461503 | 5' - CCCATTTCCGTTTTCTATTCATTG - 3'  5' - TGATTTTCCAGGCAGTCAGTCA - 3' | 54°C / 15 s |
| 212964 | 5'- GCCCATTTCATTCCATCTCATTC -3'  5'- CATCTCTTGCCTAAACCGCCT -3' | 57°C / 15 s |
| 351626 | 5'- GATCCGCTTATTAGTTTGCCTTTCGT -3'  5'- CAGCAGCACATTGACCTGACTTG -3' | 60°C / 15 s |
